# Developing a Standardized Behavior Response Team on Medical Inpatient Units to Reduce Harm

**DOI:** 10.1097/pq9.0000000000000490

**Published:** 2021-09-02

**Authors:** Michael E. Schweer, Lisa Herrmann, Marykay Duncan, Brenda Demeritt, Angela M. Statile

## Abstract

Children’s Hospitals’ Solutions for Patient Safety (SPS) is a network of over 140 children’s hospitals who share the vision of working together to eliminate serious harm across all pediatric hospitals. The SPS network is built on the fundamental belief that by sharing successes and failures transparently and learning from one another, children’s hospitals can achieve their goals more effectively and quickly than working alone. Each year, SPS hosts National Learning Sessions to which members are invited to submit abstracts describing relevant safety research or improvement work. The following abstracts were among the top submitted for the SPS Spring 2021 National Learning Session.

## Background:

Higher volumes of patients with behavioral crises admitted to medical units have led to patient and staff injuries. Staff felt ill-equipped to predict and respond to behaviors to support patients and ensure safety for all involved.

## Objectives:

To implement a standardized, multidisciplinary team response to behavioral escalation on the medical inpatient units.

## Methods:

A multidisciplinary committee performed a Failure Modes & Effects Analysis, which yielded improvement opportunities, notably the need for a team to prevent and respond to crises. Key drivers related to development of a Behavior Response Team (BRT) included: identification of roles-responsibilities, behavior de-escalation, BRT members trained in de-escalation techniques collaborating with the bedside team and medical providers, and notification process development. Two pathways were designed: (1) BRT Huddle for early signs of escalation to develop a behavioral/safety plan and (2) BRT STAT for urgent response to patients at immediate risk of harm to self or others. The BRT leader trained in de-escalation techniques utilizes a scripted process to facilitate the response and post-event debriefing.

## Results:

**Fig. 1.**
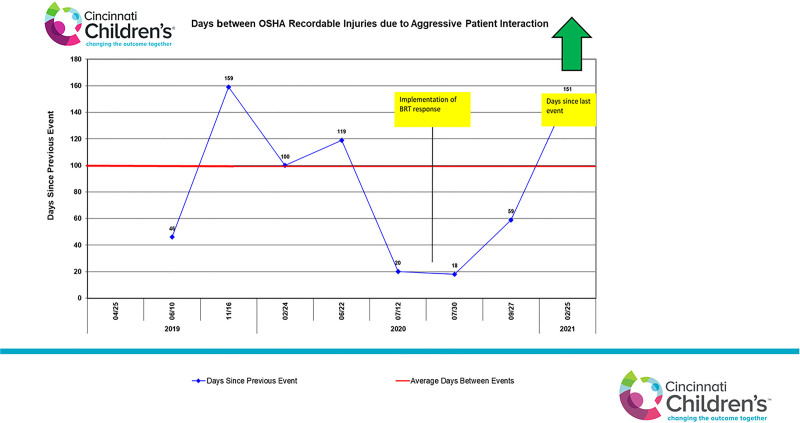
Days between OSHA-recordable injuries due to aggressive patient interaction.

The BRT was piloted on one medical unit and subsequently implemented on 14 inpatient units and the pediatric intensive care unit. BRTs have been utilized with frequency across the system since implementation. In the 7 months since implementation, 91 BRTs have been called, with 3 non-OSHA injuries. During this same time, 3 OSHA-recordable injuries occurred due to aggressive interactions, none during a BRT event. It has been 151 days since our last OSHA-recordable event (Fig. 1).

## Conclusions:

BRT implementation streamlined response efficiency and increased staff support during complex behavioral crises. Next steps include spread to other noninpatient areas.

